# When is hastened death considered suicide? A systematically conducted literature review about palliative care professionals’ experiences where assisted dying is legal

**DOI:** 10.1186/s12904-019-0451-4

**Published:** 2019-08-31

**Authors:** Sheri Mila Gerson, Amanda Bingley, Nancy Preston, Anne Grinyer

**Affiliations:** 1 School of Interdisciplinary Studies, Dumfries Campus, University of Glasgow, Dumfries, Scotland DG1 4ZL UK; 2 Division of Health Research, Lancaster University, Lancaster, UK; 3 International Observatory on End of Life Care, Division of Health Research, Lancaster University, Bailrigg, Lancaster, LA1 4YG UK

**Keywords:** Suicide, Assisted suicide, Euthanasia, Assisted dying, Hospices, Palliative care, Health personnel

## Abstract

**Background:**

Laws allowing assisted suicide and euthanasia have been implemented in many locations around the world but some individuals suffering with terminal illness receiving palliative care services are hastening death or die by suicide without assistance. This systematic review aims to summarise evidence of palliative care professionals’ experiences of patients who died by suicide or hastened death in areas where assisted dying is legal and to understand when hastened death is considered to be a suicide.

**Methods:**

AMED, CINAHL Complete, PsycINFO, PubMED, and Academic Search Ultimate were searched for articles from inception through June 2018. Quality assessment used the Hawker framework.

**Results:**

A total of 1518 titles were screened resulting in thirty studies meeting eligibility criteria for this review. Published studies about professionals’ experiences from areas with legalised assisted dying includes limited information about patients who hasten death outside legal guidelines, die by suicide without assistance, or if the law impacts suicide among palliative care patients.

**Conclusion:**

There are a range of experiences and emotions professionals’ experience with patients who die by euthanasia, assisted suicide, or hasten death without assistance. The included literature suggests improved communication among professionals is needed but does not explicitly identify when a hastened death is deemed a suicide in areas where assisted dying is practiced. More research is needed to help clarify what hastened death means in a palliative care context and identify how and if assisted dying impacts issues of suicide in palliative care settings.

**Electronic supplementary material:**

The online version of this article (10.1186/s12904-019-0451-4) contains supplementary material, which is available to authorized users.

## Background

Several jurisdictions around the world have passed assisted dying laws that allow a physician, or, in some areas, a nurse practitioner to legally prescribe and/or administer life-ending medications to individuals faced with a serious illness [1]. Some of these individuals are in receipt of palliative care services, however, they are deciding to die by suicide or hasten their death without legal lethal means. The term ‘hasten death’ is used because not all hastened deaths of people who are considered terminally ill may be considered suicide. People who intentionally hasten death have been described as individuals “who have been involved in decisions about their care” who want to have control over the circumstances of their death [2] and who may act on their wish to die with or without assistance from another individual. The desire for or wish to hasten death as a phenomenon [3] is distinguished from the act of hastened death because having a wish to die does not mean that a person will act on the desire to die [4]. The scope of literature reviews that gather evidence of professionals’ experience of caring for a patient who dies by suicide or intentionally hastens death predominantly include countries and communities where there are no laws regulating assisted dying or are limited to perspectives about the laws [5–10]. This review gathered evidence of professionals’ experiences with patients receiving palliative care services who intentionally hasten death or die by suicide in areas that allow assisted dying to understand when a hastened death is considered a suicide.

The term ‘assisted dying’ applies to various laws or court rulings currently in effect in several countries around the world including Belgium, Canada, Colombia, Luxembourg, Netherlands, Switzerland, Victoria, Australia and several jurisdictions of the United States [1]. Terms related to assisted dying have evolved around the world, including the use of the word suicide in the language of some of the laws, but it is not the intention of this review to explore the evolution of the language of the laws. The term ‘assisted suicide’ is only included in the language of the laws in Belgium, Luxembourg, and the Netherlands [11–13]. Canada, Québec, the state of Victoria in Australia, and US states that have passed assisted dying laws but do not use the term ‘suicide’ in the content of laws or court ruling. The implementation of these laws, however, are interpreted by many as a form of suicide that in some cases is considered rational for an individual who seeks assistance to die if life becomes unbearable, or if suffering from a life-threatening illness [14–16]. For the purpose of this review terms will follow the European Association of Palliative Care (EAPC) definition of assisted dying that includes euthanasia, where a medical professional administers a lethal dose of medication to a capable individual who requests it, and physician assisted suicide or assisted suicide, where a capable individual self-administers a prescribed lethal dose of medication [17]. A definition of terms used is included in Table 1.

**Table 1:** Definition of terms

Assisted suicide	Someone makes the means of death available but does not act as the direct agent of death
Euthanasia	A medical professional administers a lethal dose of medication to intentionally end a patient’s life
Hastened death	Someone who acts on their desire to control the circumstances of their death with or without assistance from another individual
Physician assisted suicide	A medical provider writes a prescription for a lethal dose of a drug to be self-administered by a competent, terminally ill patient
Suicide	Self-caused death

As of July 2019, areas of the United States that have passed assisted dying legislation that allow an individual to request a legal lethal dose of medication from a physician that can be self-administered include California, Colorado, District of Columbia, Hawaii, Maine, New Jersey, Oregon, Vermont and Washington State [18–24]. It is anticipated that more laws will be passed as jurisdictions around the world introduce and debate legalising options for capable individuals facing terminal illness to control timing of death. For example, the legislature in the state of New Jersey passed the Aid in Dying for the Terminally Ill Act [25], Victoria, Australia implemented Voluntary Assisted Dying in 2019, and New Zealand legislators are in the process of debating the End of Life Choice Bill [26, 27]. The state of Montana in the United States has passed a court ruling and Switzerland has a penal code that allow eligible individuals to request from a physician, a legal lethal dose of medication for self-administration [28, 29].

Belgium, Luxembourg, the Netherlands, and Canada allow both euthanasia (medically administered medications to cause death), and assisted suicide (self-administered medications to cause death) but Québec and Colombia only allow euthanasia.

## Method

### Review question

The question asked in this review is “When is hastened death deemed a suicide by palliative care professionals working in areas where assisted dying is legal?”

### Review design

The purpose of this literature review is to provide a comprehensive understanding of professionals’ experiences with palliative care patients who hasten their death in areas where assisted dying is legal and understand when hastened death is considered to be a suicide. Narrative synthesis is an approach to synthesising research which includes the results of both qualitative and quantitative studies [30]. This review is approached systematically and in a rigorous manner containing the six essential elements identified by Popay et al. [30]: identifying the review focus, specifying the review questions, identifying studies to include, data extraction and quality appraisal, synthesis, and dissemination. The analysis is primarily an inductive approach, and the results follow Popay et al’s [30] recommended guidelines for a narrative synthesis. This review sought research articles using data from jurisdictions where it is legal to permit assistance in dying using the EAPC definitions of euthanasia, physician assisted suicide, and assisted suicide.

### Search strategy

#### Database searches

Five electronic databases were searched from inception through to June 2018 using a strategy developed for PubMED and adapted for AMED, CINAHL, PsycINFO and Academic Search Ultimate. Searches were filtered out using Medical Subject Headings (MeSH) and some terms adapted and expanded to apply to each of the databases. Searches were conducted by SMG and each step was discussed with AG and AB. Titles and abstracts of studies were screened after duplicate citations were deleted and full text articles meeting eligibility criteria were accessed and reviewed.

Search terms were selected into four groupings. The first grouping identified terms associated with suicide. The second grouping identified terms associated with palliative care. The third grouping identified professional groups and finally the fourth group was specific to experiences. When possible, Medical Subject Headings (MeSH) terms were used. Additional file 1 includes details of the PubMED query. Table 2 identifies search terms used initially in PubMED and adapted for other databases:

**Table 2:** Database search terms

Terms	MeSH terms
1. Suicide OR assisted suicide OR assisted dying OR aid in dying OR death with dignity OR active euthanasia AND	“suicide” “suicide, assisted” “suicide, attempted” “euthanasia, active, voluntary”
2. Hospice care OR hospices OR hospice and palliative nursing OR palliative care OR palliative medicine AND	“hospice care” “hospices” “hospice and palliative nursing” “palliative care” “palliative medicine” “terminal care”
3. Professional OR clinician OR physician OR nurse OR social worker OR chaplain AND	“health personnel” “attitude of health personnel” clinician*, physician*, social worker*, chaplain*, nurse*
4. Experiences	Experience*

*used to enable different forms of a word to be searched for simultaneously increasing the number of search results found

### Selection criteria

Inclusion and exclusion criteria were developed to select the included studies (see Table 3).

**Table 3:** Inclusion and exclusion criteria

	Inclusion	Exclusion
Population	Professionals in a palliative care and hospice context: nurses, physicians, social workers, chaplains working in locations where assisted dying is allowed	Patients, public, other carers. Locations where assisted dying, including euthanasia and assisted suicide is not allowed
Intervention	Hastened Death, Euthanasia, Suicide, Assisted Suicide, Physician Assisted Suicide	
Outcomes	Professionals’ experiences with patients who died by suicide, self-administered medications prescribed by clinician, or administration of medications by clinician with intent to hasten death	Research that does not include information about direct experiences: involvement, attitudes, communication, views
Study design	Any research method/design	Opinion pieces; editorials
Reporting (language/time period)	English Data obtained from reports from inception to present	Studies conducted prior to implementation of assisted dying laws, code, or ruling of each country or state

### Assessment of quality

This review used an appraisal scale created by Hawker, et al. [31] that recognises the complexity of quality assessment from diverse research designs. The appraisal tool for this review was chosen because the questions can be used as a guideline to assess the strength of each study relevant to the research question. See Additional file 2 for the version of Hawker, et al. [31] question and rating system. Each study was given a score between 9 and 36 based on a series of questions. No studies were excluded from this review due to quality.

### Data extraction and method of synthesis

Data collected from the literature included study methods, participants, sample size, setting, research focus, and terminology used about assisted dying. Data were extracted and included in a table of characteristics identified by SMG and reviewed with AG and AB. SMG then analysed the studies for themes discussing the results with AG and AB. Popay et al. [30] suggests narrative synthesis is a way of telling the story of research evidence that is also flexible when combining studies from diverse methodologies that seemed most appropriate for this review.

### Overview of studies

**Fig. 1:**
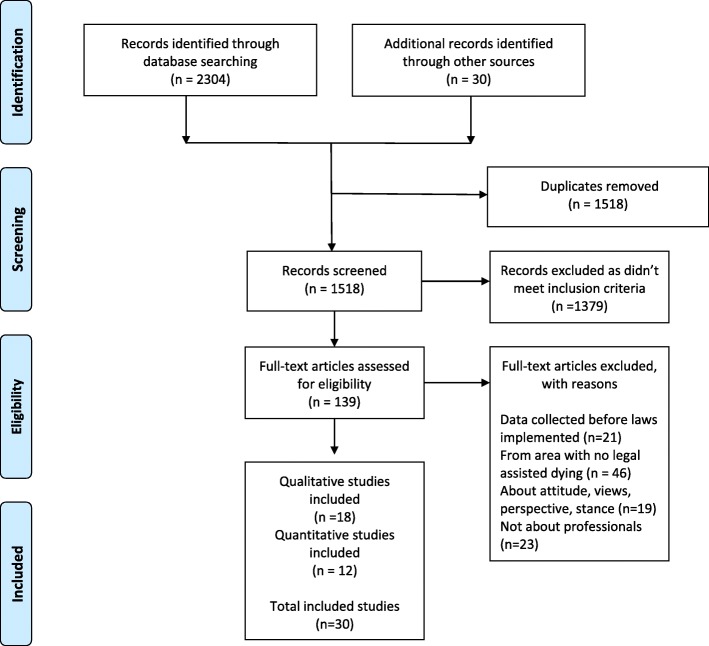
PRISMA flow diagram of study selection

A total of 30 studies were selected as meeting the inclusion criteria for this review. References of the full text articles were searched using Google Scholar and an additional 30 full text articles were included for consideration. SMG screened full text articles to determine eligibility for final inclusion in the review. Duplicates were deleted and abstracts then screened against the inclusion criteria before considering study quality. An adapted version of the Preferred Reporting Items for Systematic Reviews and Meta-Analyses (PRISMA) flowchart in Fig. 1 summarises how the 30 articles were identified for the review:

A total of 30 studies were included in this review. Of these studies:

- Five studies are from Belgium, nine from the Netherlands, two from Switzerland, 13 from the United States, and one is from Canada.
- Only three of the 30 studies include information about experiences of hastened death that is not assisted suicide (self-administered lethal dose of medication) or euthanasia (lethal medication injected by professional); Ganzini et al. [32] investigate the phenomenon of patients who voluntarily refuse food and fluids, and Anquinet et al. [33] compare euthanasia with continuous deep sedation.
- Two studies are part of a larger survey about the role of Dutch nurses in euthanasia and physician-assisted suicide [34, 35].
- Four papers are reported from one survey of physicians in the United States [36–39].
- Four papers are from the data results of one study of hospice workers [32, 40–42].

The professionals in the included studies were primarily limited to physicians and nurses, with four studies from the United States that included social workers and one survey of chaplains [40–44].

## Results

The 30 studies are summarised in Table 4. Analysis of these studies identified four primary themes:

**Table 4:** Characteristics of studies

Reference	Participants	Study Methods	Data Year	Research Focus	Setting	Terminology Used in Study
Belgium						
Anquinet L et al. (2013) [33]	Two groups of physicians (*n* = 8) and two groups of nurses (*n* = 13)	Qualitative focus groups	2010	Similarities and differences between the practice of continuous sedation until death and the practice of euthanasia.	Homecare and hospital setting-palliative care unit support team	Physician Assisted Death; Euthanasia
Cohen J et al. (2012) [45]	Physicians 480-Flanders; 305-Wallonia	Postal survey	2008 and 2009	Cultural differences between Walloon and Flemish physicians affecting euthanasia practice	Unspecified	Euthanasia
Dierckx De Casterlé, et al. (2010) [46]	18 nurses	Qualitative in-depth interviews	2005–2006	Nursing care for patients requesting euthanasia in general hospitals in Flanders, Belgium	Included palliative care units	Euthanasia
Inghelbrecht, E., et al. (2010) [47]	1678 nurses	Second phase of two-phase postal survey	2007–2008	Nurses involvement inconsistent in decisions about euthanasia or drugs to end a patients’ life, and nurses administer life-ending drugs even though it is not regulated	Homecare, care homes, hospital	Euthanasia by patients’ request; Administration of life ending medications without patients’ explicit request
Meeussen, K., et al. (2011) [48]	205 GPs	Structured phone survey	2005–2006	Investigated how many patients expressed a wish for euthanasia, documentation of these requests, experiences with these requests, and how it was handled	Focused on patients who died at home	Euthanasia
Canada						
Beuthin, R., et al. (2018) [49]	17 nurses	Qualitative interviews	2016	Explored nurses experiences of MAiD	Hospital, hospice, home settings	Medical Assistance in Dying (MAiD), euthanasia, assisted suicide
Netherlands						
Dees, M. K., et al. (2013) [50]	32 cases, 31 relatives, 28 physicians	Qualitative interviews	2008–2009	Explores decision-making process in cases where patients request euthanasia	Homes or physicians’ offices	Euthanasia
Georges, J. J., et al. (2008) [51]	30 GPs	Qualitative in-depth interviews	2002–2003	Describes experiences of general practitioners (GPs) in Netherlands dealing with a request for euthanasia from a terminally ill patient.	Unspecified	Euthanasia
Francke, A. L., et al. (2016) [52]	587 respondents (65%) Nurses and CNAs	Postal survey	2011	To give insight into Dutch nursing staff’s attitudes and involvement regarding euthanasia	Unspecified	Euthanasia
Norwood, F. (2007) [53]	10 physicians included in study	Ethnographic study: Foucauldian discourse	2001	Euthanasia, general practice, and end-of-life discourse in the Netherlands	Home	Euthanasia
Pasman, H. R., et al. (2013) [54]	11 physicians and 9 patients, 3 relatives	Qualitative Interviews	2005–2007	What happens after request for euthanasia is refused	Unspecified	Euthanasia or Physician Assisted Suicide (EAS)
Snijdewind, M. C., et al. (2014) [55]	28 physicians 26 relatives	Qualitative in-depth interviews	November 2011–April 2012	According to the physicians and relatives, what are the characteristics of the complexities that can arise from the moment someone requests EAS?	Unspecified	Physician Assisted Suicide; Euthanasia
Van Bruchem-van de Scheur, G., et al. (2007) [35]	500 district nurses-86% response rate. 81.6% (408) used for analysis	Postal survey	2003	Euthanasia and physician assisted suicide in the Dutch home care sector: the role of the district nurse	55 home care organisations	Physician Assisted Suicide; Euthanasia
Van Bruchem-van de Scheur, G.,et al. (2008) [34]	1509 nurses; 1179 responses analysed (78.1%)	Telephone survey	2003	The role of nurses in euthanasia and physician-assisted suicide in the Netherlands	Hospital, nursing homes and home care	Physician Assisted Suicide; Euthanasia
Van Marwijk, H., et al. (2007) [56]	22 physicians	Qualitative, four focus groups	2003	Impact of euthanasia on primary care physicians in the Netherlands	Primary care	Euthanasia; Assisted Suicide
Switzerland						
Otte, I. C., et al. (2016) [57]	23 General Practitioners (GPs)	Qualitative interviews	2012–2013	Barriers to professional communication about Physician Assisted Suicide	Unspecified	Physician Assisted Suicide
Gamondi, C., et al. (2017) [28]	23 palliative care physicians	Qualitative study	2015	Physician responses to assisted suicide requests	Unspecified	Assisted Suicide
United States						
Carlson, B, et al. 2005 [43]	50 out of 77 hospice chaplains (65% response)	Postal survey	2003	Attitudes and experiences of hospice chaplains with patients requesting physician assisted suicide	Hospice home care	Physician-Assisted Suicide (PAS); Oregon Death with Dignity Act (ODDA)
Chin, et al. 1999 [58]	14 physicians	Qualitative interviews	Report of 1998 data	First year’s experience with legalised assisted suicide in Oregon	Unspecified	Physician-Assisted Suicide
Clymin, J., et al. 2012 [59]	582 out of 7500 nurses	Qualitative component of email survey	2010	Nurses knowledge of the Death with Dignity Act	Unspecified	Death With Dignity Act (DWDA) Physician Assisted Death (PAD)
Dobscha, SK, et al. 2004 [36]	35 physicians	Qualitative semi-structured interviews	2000	Physicians responses to requests for assisted suicide	Unspecified	DWDA; Assisted Suicide
Ganzini, L., et al. 2003 [37]	35 physicians	Qualitative in-depth interviews	2000	Oregon physicians’ perceptions of patients who request assisted suicide	Unspecified	Assisted Suicide
Ganzini, L., et al. 2000 [39]	2649 of 4053 physicians (65% response)	Postal survey	1999	Physicians’ experiences with the Oregon Death with Dignity Act	Unspecified	Oregon Death with Dignity Act
Ganzini, L., et al. 2001 [38]	2641 physicians (65% response)	Postal survey	1999	Physicians’ attitudes/experiences with end-of-life care since passage of the Oregon Death with Dignity Act	Unspecified	DWDA
Ganzini, L., et al. 2002 [40]	307 nurses and social workers of 545 (72–78% response)	Postal survey	2001	Experiences of nurses and social workers with hospice patients who requested suicide assistance	Hospice home care	Assisted Suicide
Ganzini, L., et al. 2003 [32]	307 nurses of 429 (72% response)	Postal survey	2001	Nurses’ experiences with hospice patients who refuse food and fluids to hasten death	Hospice home care	Voluntary Refusal of Food and Fluids (VRFF) Physician Assisted Suicide (PAS)
Harvath, et al. 2006 [41]	20 nurses and social workers	Qualitative interviews	Not clearly stated	Dilemmas encountered by hospice workers when patients wish to hasten death	Hospice home care	Physician Assisted Suicide; Hastened Death
Miller, L. et al. 2004 [42]	306 nurses and 85 social workers (72–78% response)	Postal survey	2001	Attitudes/experiences of Oregon hospice nurses and social workers regarding assisted suicide	Hospice home care	Assisted Suicide; Oregon Death with Dignity Act (ODDA)
Norton, E. M. & Miller, P. J. 2012 [44]	9 social workers	Focus group	Since passing of the law	Social workers discuss DWDA	Hospice home care	DWDA
International (Netherlands and Oregon)						
Voorhees, J. R., et al. (2014) [61]	36 physicians	Qualitative semi-structured interviews	2007–2008	Physicians’ experiences in the United States and the Netherlands	No	Physician-Assisted Dying (PAD)

1. Assisted suicide is frequently not distinguished from broader instances of suicide
2. VSED and Sedation are seen as alternative to assisted dying
3. A broad knowledge of hastened death and suicide to assess and support patients
4. Communication challenges and opportunities

Each of these themes is described below:

### Assisted suicide is frequently not distinguished from broader instances of suicide

Authors frequently refer to suicide when they are speaking about physician assisted suicide.

There is almost no literature about patients who die by unassisted suicide in hospice and palliative care services in areas that allow assisted dying. Deaths by suicide are not identified and differentiated from medically assisted deaths. What is termed physician assisted suicide (PAS), might also be called Physician Aid in Dying or Physician Assisted Death (PAD), or only ‘assisted suicide’ in some studies. Only one study from The Netherlands identified patients who died by unassisted suicide, attempted an unsuccessful overdose of medications, or deliberately stopping eating and drinking when their request for euthanasia was refused [54]. In Switzerland, the word ‘physician’ is removed from the term ‘assisted suicide’ because physicians can only be present as a private citizen at the time the patient ingests lethal medications [28].

Most of the included studies from the United States use the word ‘suicide’ in the title or in the content of their research. In research from the United States authors often used the unqualified term ‘suicide’ when discussing physician-assisted suicide [36, 39, 43, 59]. For example, Carlson et al. [43], state hospice policies may prevent “employees from assisting the patient in obtaining the prescription or attending a suicide” (p. 1164), suggesting that patients who utilise the legally prescribed lethal medication, die by suicide. Authors of the included studies imply patients who request their physicians to legally prescribe lethal medications and then use these medications to end their life, end their life through suicide [36, 39, 59].

Most authors acknowledge terminology variation but do not explain their choice of terms around this topic. Some authors refer to terms used in legislation such as ‘aid in dying,’ ‘legalised physician assisted suicide,’ ‘PAS,’ ‘assisted suicide,’ or the ‘Oregon Death with Dignity Act’ [34, 35, 39, 55, 57]. Other authors choose ‘physician-assisted death’ or ‘physician–assisted dying’ as a capture term to include both euthanasia and assisted suicide instead of the terms ‘physician-assisted suicide’ or ‘assisted suicide’ [61]. Gamondi, et al. [28] acknowledge that there is controversy over the terminology and use ‘assisted dying’ as a neutral term to describe both euthanasia and assisted suicide.

### VSED and sedation alternative to assisted dying

Voluntary Refusal of Food and Fluids (VRFF), also known as Voluntary Stopping of Eating and Drinking (VSED), and continuous deep sedation are sometimes characterised as hastened death and as alternatives to assisted dying [32, 33]. There is a variation of experiences and whether they are considered acceptable. Ganzini et al’s [40], study of nurses and social workers report VRFF as an alternative option for patients who seek physician assisted suicide but do not identify it as a form of suicide. A study of social workers in the United States and a study with physicians from The Netherlands were the only ones to identify ‘suicide’ as separate from other forms of hastened death, suggesting suicide occurs separately from assisted dying and VRFF [44, 54].

### A broad knowledge of hastened death and suicide to assess and support patients

This theme is separated into two subthemes: assessing patients and determining eligibility, and fear and conflict. First, professionals’ have a broad range of experiences that include assessing and determining eligibility for assisted dying. Second, some experiences of different professional groups, suggest personal feelings of fear and conflict being involved in the assessment process of working with patients who are seeking to hasten death.

#### Assessing patients and determining eligibility

The terms ‘mentally competent’ or ‘mentally capable’ are frequently used to determine whether a patient is eligible to get lethal medications from a physician. The literature suggests, however, that physicians are not alone in determining eligibility and assessing patients for the option for a legal lethal dose of medications. Different professionals discern and address these issues in diverse ways depending on the policy of their employer, institutional or personal interpretation of suicide, and sense of responsibility for the patient [59]. Several authors indicate that organisational policy presents dilemmas around needing to prevent hastened death, but most speak of the critical need to establish clear guidelines for interdisciplinary team members [34, 41, 46]. These dilemmas include concern that nurses lacked knowledge and training about policy and legislation potentially causing patients to receive misinformation about options [59]. Overall, most nurses in these studies indicate they would support a patients’ choice but did not address nurses’ experience with assessing patients who died by suicide other than through legally prescribed medication by a physician or by VRFF [32, 59].

#### Fear and conflict

The studies of physicians included in this review describe a range of emotions and experiences about responding to requests for medications to end patients’ lives. These emotions range from feelings of fear, frustration, and isolation to feelings that their experience improved their relationship with patients, and increased quality of life and comfort when writing prescriptions or administering lethal medications [38, 56–58, 61]. Some literature about physicians’ experiences associates feelings of anxiety and fear with the word ‘suicide’ as the physician struggles to assess the risk and needs of their patients [32, 36]. The span of emotions demonstrated by these studies suggest an increased sense of responsibility for patients compared to other professionals, which may be attributed to the power they possess to prescribe lethal medications to control timing of death, and from the physicians’ concerns about being able to provide comfort until death [51]. In addition, it is important to note that palliative care emerged emphasizing comfort rather than hastening of death [62, 63]. The doctrine of double effect [64] may have influenced and increased fear and frustration among physicians who were trying to promote and educate about palliative care while struggling with patient requests to hasten death.

Norton and Miller’s study about hospice social workers in Oregon [44], suggests that patients save up medications to have the option to end their own life and do, at times, successfully die by suicide but does not explicitly state their role in assessment for assisted dying or for suicide risk. Other studies explore the complexity of the social worker role in understanding and identifying when a patient is at risk of suicide and when compared with nurses, indicate that social workers are more accepting of patient choices [40].

Nurses experience fear and ethical conflict, particularly when questions posed by patients about access to lethal medications conflicted with their own personal or religious beliefs but feel increased acceptance when patients make the decision to stop eating and drinking [32, 49]. Harvath et al. [41] suggest that hospice nurses understood that patient choice to control circumstances of death may be more important than other issues of concern.

Inghelbrecht et al. [47], however, do examine the incidence of nurses administering life-ending medications with and without a patient’s explicit request for hastened death, despite nurses’ direct administration being unacceptable and unregulated by Belgium’s euthanasia law. There is little indication from other studies that nurses participate in euthanasia with or without the patient’s consent. Beuthin et al. [49] emphasise the moral distress some nurses experience particularly when a patients choice to die is in conflict with their own beliefs.

There is only one study about chaplains indicating that whether or not chaplains agree with a patients’ decision to hasten death, they support patients’ choices. Carlson suggested that chaplains working with patients interested in pursuing the DWDA did not influence the decision to hasten death [43]. This study was not clear about the chaplains role or influence in assessing patients for assisted dying eligibility.

Professionals have a range of experiences that may be attributed to cultural differences, as explained by Cohen et al., in a study from Belgium [45]. Cohen et al. write that “the influence” of a law “on a particular society... is affected by the surrounding culture” (p. 852). Understanding cultural differences, including language, religion, and environment may be a key element to understand and interpret experiences around issues of assessing suicide risk and eligibility for assisted suicide.

### Communication challenges and opportunities

A theme identified in the literature is that there are both challenges and opportunities to speak openly about issues of suicide among professionals of the same or different disciplines and between professionals and those they serve [36, 50, 52–54, 57]. The included articles suggest that with assisted dying there is a tendency to view professionals’ conversations with patients as opportunities to discuss what is relevant and important to the patient, thus improving end-of-life care. Studies of physicians assert that passage of the laws is associated with an increased sense of responsibility to provide optimal palliative care and serves as an opening for communication on important end of life issues [61]. Ganzini et al. [38] suggest that since passage of the DWDA in Oregon, physicians have increased referrals and access to hospice care, but relationships may have been altered when a physician does not agree to prescribe lethal medications. A Swiss study of physicians indicates that while few agree to prescribe lethal medications they assume a responsibility to discuss the origin of the patient’s request and seek alternatives to assisted suicide [28].

Studies of other professionals corroborate the research about physicians indicating “the discussion about assisted suicide opens up the box for them to ask all of the other questions they have...” [41]. Some research, however, indicates that communication with patients is impaired when there is understanding that organisational policy, professional code, or lack of training restricts professionals from communicating about options for care [59]. Several authors discuss the need for more training [33, 54, 57]. The implication of most of these studies is that professionals other than physicians are involved in determining eligibility for assisted dying options and suggest the importance of education, and team communication about legal options for patients.

## Discussion

### Main findings

This review finds that professionals’ experiences of hastened death and suicide with palliative care patients in areas where assisted dying is authorised are primarily focused on legalised self-administered or clinician administered lethal medications. Overall, there is little clarity on the question of whether experiences of hastened death are deemed suicide. In addition, there is little understanding about patients who use other methods to end their life with or without assistance in areas where assisted dying is legal. Suicide implies that a person has an intention for self-harm resulting in death; however, research suggests that the definition of suicide depends on who is doing the research and how the research is completed [65]. Whether the pursuit of assisted dying is considered suicide is debatable and may depend on culture or understanding of the law.

Finally, the recommendations of the included studies suggest further research is needed as more jurisdictions and nations take steps to legalise assisted dying. These recommendations include the need to explore organisational policy affecting professionals’ ability to provide information to patients, differences between assisted dying, voluntary refusal of food or fluids, or continuous deep sedation, and the need to increase information to provide guidance for healthcare professionals working with patients who report a desire to hasten death [28, 41, 46, 54, 59]. The review findings show that there is great variance in nomenclature and definitions that require careful analysis and are important in identifying professionals’ work with individuals facing fear, pain, and suffering at end of life. The broader review of literature indicates widespread international engagement with these issues.

### Limitations

There were limitations to this review that deserve mention. First, the search and synthesis were conducted by the primary author (SMG) which increases the potential for bias and error. The terms used to describe assisted dying in this study is further influenced by the definition of the EAPC that may not be shared by all groups or professional associations.

The study did not include other professionals including pharmacists, psychiatrists, or nursing assistants who have important roles working with patients receiving palliative care. Studies that included a mental health or psychiatric perspective were excluded because they were not specifically focused on experiences within palliative care [60]. Mental health professionals and psychiatrists, however, have a unique role working with individuals seeking an assisted death focused on competency assessment and eligibility that warrants further study. The review was limited to literature focused on patients receiving palliative care and not on literature of patients facing advanced illness such as cancer, which may have added additional insight into knowledge about individuals who killed themselves in areas where assisted dying is allowed.

Some studies varied in quality and were limited in scope containing brief conclusions about the topic [58]. Other studies were completed in areas where assisted dying had been implemented for many years compared to a more recent study, such as the study of nurses from Canada when assisted dying had only been available for 6 months [49]. Several jurisdictions have implemented assisted dying laws in the United States since 2008 yet most studies conducted in the United States were focused in Oregon. The variation in laws around the world, and integration of palliative care practice with assisted dying may be different in each of these jurisdictions. This review is relevant in areas where there is legalised assisted dying, but the included studies do not lead to a firm conclusion. In addition, the larger historical context, evolution and implementation of the laws, mollification of the term suicide in the content of the laws and different types of assisted dying practices are not the specific focus of this study, but worthy of future research.

### Strengths

This review was conducted systematically and with a rigorous approach. The systematic search and data extraction are replicable. The review question addresses an area of research that has not been done previously and contributes critical information about caring for individuals at the end of life. Both quantitative and qualitative studies demonstrated the paucity of evidence directly relevant to the research question. The strength of these findings has the potential to impact palliative care assessment and practise in clinical, hospital and home settings. Including all of the relevant studies in the synthesis enabled a broader understanding of the interpretation and terms used in the literature.

## Conclusion

The aim of this review was to answer the research question about when hastened death is deemed a suicide by palliative care professionals working in areas where assisted dying is legal. The results indicate that it is unclear how and when a patients’ death is considered a suicide, whether the patient dies from an overdose of medications, utilises assisted dying, or hastens death unassisted. Overall, this review demonstrates that significant gaps remain in the literature regarding hastened death, assisted dying and suicide with patients receiving palliative care. Understanding professionals’ experiences with patients who die by suicide can contribute to improving communication and assessment with patients around issues of hastened death and provide needed guidance for future research in all jurisdictions. However, the literature identified in this review indicates that there is limited information and research about professionals’ experiences of patient suicide in areas that allow assisted dying. Improved knowledge about palliative care team experiences can improve communication about issues of suicide and alleviate individual fear over what has been considered by many to be a taboo topic [66]. If open discussion about death is considered taboo, then suicide is likely to trigger even deeper anxiety and fear within that taboo of death [67]. If palliative care is indeed patient-centred, then opportunity for open conversation about whatever is important to the patient must be available.

## Additional files

Additional file 1 PubMed Query. (DOCX 15 kb)

Additional file 2 Study Quality Form. (DOCX 16 kb)

## Data Availability

All data and materials supporting the data and conclusions are available on request.

## Abbreviations

DWDA: Death with Dignity Act
EAPC: European Association of Palliative Care
MeSH: Medical Subject Headings
PAD: Physician Assisted Death
PAS: Physician assisted suicide
PRISMA: Preferred Reporting Items for Systematic Reviews and Meta-Analyses
VRFF: Voluntary Refusal of Food and Fluids
VSED: Voluntary Stopping of Eating and Drinking
