# Supplementary material for: When is hastened death considered suicide? A systematically conducted literature review about palliative care professionals’ experiences where assisted dying is legal
Source: BMC Palliat Care. 2019 Aug 31;18:75. doi: 10.1186/s12904-019-0451-4 (PMC6717643; doi:10.1186/s12904-019-0451-4)
Supplement: Supplementary file 1 — PubMed Query. (DOCX 15 kb) [file 12904_2019_451_MOESM1_ESM.docx]

Additional file 1: PubMed Query

| 10 July 2018 Query - Pubmed | |  |
| --- | --- | --- |
| Search | Query | Items found |
| #12 | Search ((((((((("suicide"[MeSH Terms]) OR "suicide, assisted"[MeSH Terms]) OR "suicide, attempted"[MeSH Terms]) OR "euthanasia, active, voluntary"[MeSH Terms])) OR (((("assisted suicide"[Title/Abstract]) OR "assisted dying"[Title/Abstract]) OR "aid in dying"[Title/Abstract]) OR "death with dignity"[Title/Abstract]))) AND (((((((("hospice care"[MeSH Terms]) OR "hospices"[MeSH Terms]) OR ("hospice and palliative care nursing"[MeSH Terms])) OR "palliative care"[MeSH Terms]) OR "palliative medicine"[MeSH Terms])) OR "terminal care"[MeSH Terms]) OR (("end of life"[Title/Abstract]) OR hospice*[Title/Abstract]))) AND (("health personnel"[MeSH Terms]) OR ((((((professional*[Title/Abstract]) OR clinician*[Title/Abstract]) OR physician*[Title/Abstract]) OR nurse*[Title/Abstract]) OR social worker*[Title/Abstract]) OR chaplain*[Title/Abstract]))) AND experience*[Title/Abstract] | 297 |
| #11 | Search ((((((((("suicide"[MeSH Terms]) OR "suicide, assisted"[MeSH Terms]) OR "suicide, attempted"[MeSH Terms]) OR "euthanasia, active, voluntary"[MeSH Terms])) OR (((("assisted suicide"[Title/Abstract]) OR "assisted dying"[Title/Abstract]) OR "aid in dying"[Title/Abstract]) OR "death with dignity"[Title/Abstract]))) AND (((((((("hospice care"[MeSH Terms]) OR "hospices"[MeSH Terms]) OR ("hospice and palliative care nursing"[MeSH Terms])) OR "palliative care"[MeSH Terms]) OR "palliative medicine"[MeSH Terms])) OR "terminal care"[MeSH Terms]) OR (("end of life"[Title/Abstract]) OR hospice*[Title/Abstract]))) AND (("health personnel"[MeSH Terms]) OR ((((((professional*[Title/Abstract]) OR clinician*[Title/Abstract]) OR physician*[Title/Abstract]) OR nurse*[Title/Abstract]) OR social worker*[Title/Abstract]) OR chaplain*[Title/Abstract]))) AND experience*[Title/Abstract] | 1233656 |
| #10 | Search ((((((((("suicide"[MeSH Terms]) OR "suicide, assisted"[MeSH Terms]) OR "suicide, attempted"[MeSH Terms]) OR "euthanasia, active, voluntary"[MeSH Terms])) OR (((("assisted suicide"[Title/Abstract]) OR "assisted dying"[Title/Abstract]) OR "aid in dying"[Title/Abstract]) OR "death with dignity"[Title/Abstract]))) AND (((((((("hospice care"[MeSH Terms]) OR "hospices"[MeSH Terms]) OR ("hospice and palliative care nursing"[MeSH Terms])) OR "palliative care"[MeSH Terms]) OR "palliative medicine"[MeSH Terms])) OR "terminal care"[MeSH Terms]) OR (("end of life"[Title/Abstract]) OR hospice*[Title/Abstract]))) AND (("health personnel"[MeSH Terms]) OR ((((((professional*[Title/Abstract]) OR clinician*[Title/Abstract]) OR physician*[Title/Abstract]) OR nurse*[Title/Abstract]) OR social worker*[Title/Abstract]) OR chaplain*[Title/Abstract]))) AND experience*[Title/Abstract] | 98311 |
| #9 | Search ((((((((("suicide"[MeSH Terms]) OR "suicide, assisted"[MeSH Terms]) OR "suicide, attempted"[MeSH Terms]) OR "euthanasia, active, voluntary"[MeSH Terms])) OR (((("assisted suicide"[Title/Abstract]) OR "assisted dying"[Title/Abstract]) OR "aid in dying"[Title/Abstract]) OR "death with dignity"[Title/Abstract]))) AND (((((((("hospice care"[MeSH Terms]) OR "hospices"[MeSH Terms]) OR ("hospice and palliative care nursing"[MeSH Terms])) OR "palliative care"[MeSH Terms]) OR "palliative medicine"[MeSH Terms])) OR "terminal care"[MeSH Terms]) OR (("end of life"[Title/Abstract]) OR hospice*[Title/Abstract]))) AND (("health personnel"[MeSH Terms]) OR ((((((professional*[Title/Abstract]) OR clinician*[Title/Abstract]) OR physician*[Title/Abstract]) OR nurse*[Title/Abstract]) OR social worker*[Title/Abstract]) OR chaplain*[Title/Abstract]))) AND experience*[Title/Abstract] | 58518 |
| #8 | Search experience*[Title/Abstract] | 932452 |
| #7 | Search (((((professional*[Title/Abstract]) OR clinician*[Title/Abstract]) OR physician*[Title/Abstract]) OR nurse*[Title/Abstract]) OR social worker*[Title/Abstract]) OR chaplain*[Title/Abstract] | 951032 |
| #6 | Search "health personnel"[MeSH Terms] | 461971 |
| #5 | Search ("end of life"[Title/Abstract]) OR hospice*[Title/Abstract] | 27876 |
| #4 | Search "terminal care"[MeSH Terms] | 47356 |
| #3 | Search (((("hospice care"[MeSH Terms]) OR "hospices"[MeSH Terms]) OR ("hospice and palliative care nursing"[MeSH Terms])) OR "palliative care"[MeSH Terms]) OR "palliative medicine"[MeSH Terms] | 56303 |
| #2 | Search ((("assisted suicide"[Title/Abstract]) OR "assisted dying"[Title/Abstract]) OR "aid in dying"[Title/Abstract]) OR "death with dignity"[Title/Abstract] | 3772 |
| #1 | Search ((("suicide"[MeSH Terms]) OR "suicide, assisted"[MeSH Terms]) OR "suicide, attempted"[MeSH Terms]) OR "euthanasia, active, voluntary"[MeSH Terms] | 57671 |
